# Cardiac structure and function are altered in type 2 diabetes and Non-alcoholic fatty liver disease and associate with glycemic control

**DOI:** 10.1186/s12933-015-0187-2

**Published:** 2015-02-13

**Authors:** Sophie Cassidy, Kate Hallsworth, Christian Thoma, Guy A MacGowan, Kieren G Hollingsworth, Christopher P Day, Roy Taylor, Djordje G Jakovljevic, Michael I Trenell

**Affiliations:** Institute of Cellular Medicine, Newcastle University, Newcastle upon Tyne, UK; Department of Cardiology, Freeman Hospital, Newcastle upon Tyne, UK; Institute of Genetic Medicine, Newcastle University, Newcastle upon Tyne, UK; Newcastle Magnetic Resonance Centre, Institute of Cellular Medicine, Newcastle University, Newcastle upon Tyne, UK

**Keywords:** Type 2 diabetes, Non-alcoholic fatty liver disease, Cardiac disease

## Abstract

**Background:**

Both non-alcoholic fatty liver disease (NAFLD) and Type 2 diabetes increase the risk of developing cardiovascular disease. The metabolic processes underlying NAFLD and Type 2 diabetes are part of an integrated mechanism but little is known about how these conditions may differentially affect the heart. We compared the impact of NAFLD and Type 2 diabetes on cardiac structure, function and metabolism.

**Methods:**

19 adults with Type 2 diabetes (62 ± 8 years), 19 adults with NAFLD (54 ± 15 years) and 19 healthy controls (56 ± 14 years) underwent assessment of cardiac structure, function and metabolism using high resolution magnetic resonance imaging, tagging and spectroscopy at 3.0 T.

**Results:**

Adults with NAFLD and Type 2 diabetes demonstrate concentric remodelling with an elevated eccentricity ratio compared to controls (1.05 ± 0.3 *vs.* 1.12 ± 0.2 *vs.* 0.89 ± 0.2 g/ml; p < 0.05). Despite this, only the Type 2 diabetes group demonstrate significant systolic and diastolic dysfunction evidenced by a reduced stroke index (31 ± 7*vs.* controls, 38 ± 10, p < 0.05 ml/m^2^) and reduced E/A (0.9 ± 0.4 *vs*. controls, 1.9 ± 1.4, p < 0.05) respectively. The torsion to shortening ratio was higher in Type 2 diabetes compared to NAFLD (0.58 ± 0.16 *vs.* 0.44 ± 0.13; p < 0.05). Significant associations were observed between fasting blood glucose/HbA1c and diastolic parameters as well as the torsion to shortening ratio (all p < 0.05). Phosphocreatine/adenosine triphosphate ratio was not altered in NAFLD or Type 2 diabetes compared to controls.

**Conclusions:**

Changes in cardiac structure are evident in adults with Type 2 diabetes and NAFLD without overt cardiac disease and without changes in cardiac energy metabolism. Only the Type 2 diabetes group display diastolic and subendocardial dysfunction and glycemic control may be a key mediator of these cardiac changes. Therapies should be explored to target these preclinical cardiac changes to modify cardiovascular risk associated with Type 2 diabetes and NAFLD.

## Background

Type 2 diabetes effects ~5% of Western populations, with prevalence rates significantly higher in East and South Asian communities [1]. Non-alcoholic fatty liver disease (NAFLD) is reported to effect between 20 and 30% of Western populations [2], can be as high as 60% in urban East and South Asian groups [3], and is closely related to the development of Type 2 diabetes [4]. Indeed, in excess of 90% of obese people with Type 2 diabetes have NAFLD [5]. The strong relationship between NAFLD and Type 2 diabetes lies in the central role of the liver lipids in glucose homeostasis [6].

Heart disease is the leading cause of morbidity and mortality in *both* Type 2 diabetes and NAFLD [7,8]. Individuals with diabetes demonstrate a 74% greater risk of hospitalisation due to heart failure [9]. NAFLD, characterized by elevated serum γ-glutamyltransferase (GGT), is independently associated with heart failure [10]. The increased incidence of cardiovascular morbidity and mortality associated with Type 2 diabetes and NAFLD, has been linked to preclinical changes in cardiac structure, function and metabolism.

The most commonly reported change in asymptomatic individuals with Type 2 diabetes is diastolic dysfunction [11-14], alongside decreased end-diastolic blood volume [11], and changes in cardiac strain patterns [15]. These cardiac changes have been associated with myocardial steatosis [16], michondondrial dysfunction [17], changes in calcium regulation, and myocardial fibrosis [18]. NAFLD is also characterized by a similar pattern of diastolic dysfunction [19,20], altered left ventricular geometry [19], reduced myocardial perfusion reserve [21] and changes in cardiac strain [22]. Although a growing body of epidemiological and clinical evidence links the disease processes of Type 2 diabetes and NAFLD, little is known about how these conditions may differentially affect the heart.

Using magnetic resonance imaging (MRI) we have previously shown pre-clinical changes in cardiac structure and function in NAFLD [22]. To extent this work and in light of the importance of understanding early cardiac changes and reducing cardiovascular risk in people with metabolic disease, the present study was designed to compare the impact of Type 2 diabetes and NAFLD upon cardiac structure, function and metabolism and to identify potential metabolic mediators.

## Methods

### Participants

In a case control study, 19 participants with NAFLD and 19 participants with Type 2 diabetes were recruited into the study through Newcastle upon Tyne Hospitals NHS Foundation Trust. NAFLD was defined as >5% intrahepatic lipid on ^1^H-magnetic resonance spectroscopy of the liver (see below for detailed methods) with no evidence of advanced fibrosis (mean alanine aminotransferase / aspartate aminotransferase (AST/ALT) 0.82 ± 0.08). Patients with >5% intrahepatic lipid were excluded from the NAFLD group if they had a previous diagnosis of Type 2 diabetes, were on any glucose lowering medication, had an HbA1c ≥48 mmol/mol or had any secondary causes of hepatic steatosis as listed in [23]. Type 2 diabetes participants had been previously diagnosed by their GP (diagnosis length 5 ± 4 years) and were diet or metformin controlled only. Both groups had no previous history of cardiac disease. 19 controls, matched for gender, were recruited from advertisements in local newspapers and were without hypertension, metabolic, liver or cardiac disease. Participant characteristics are presented in Table 1.

**Table 1:** **Demographic and metabolic characteristics of study population by group**

**Variable**	**Controls**	**NAFLD**	**Type 2 diabetes**	**P value**
	**(n = 19)**	**(n = 19)**	**(n = 19)**	
Age (yr)	56 ± 14	54 ± 15	62 ± 8	0.127
Gender (men:women)	11:8	11:8	11:8	-
Height (cm)	169 ± 11	169 ± 9	168 ± 9	0.877
Weight (kg)	78 ± 11	83 ± 14	91 ± 14*	0.013
BMI (kg/m^2^)	28 ± 4	29 ± 3*	33 ± 5*	0.012
Body surface area (m^2^)	1.9 ± 0.2	1.9 ± 0.2	2.0 ± 0.2*	0.018
Visceral adipose tissue (cm^2^)	-	154 ± 47	191 ± 75	0.120
Systolic blood pressure (mmHg)	131 ± 11	146 ± 16*	145 ± 17*	0.003
Diastolic blood pressure (mmHg)	82 ± 8	90 ± 12	89 ± 12	0.096
VO_2peak_ (ml min^−1^ kg^−1^)	-	24 ± 6	19 ± 5†	0.007
Fasting Glucose (mmol/L)	5.2 ± 0.5	5.0 ± 0.6	7.2 ± 1.4*^†^	0.000
HbA_1c_ (mmol/mol)	-	38 ± 5	58 ± 10†	0.000
(%)		(5.6 ± 0.4)	(7.4 ± 0.9)	
Intrahepatic lipid (%)	2.5 ± 0.9	9.4 ± 4.3*	7.9 ± 6.7*	0.000
ALT (U.L)	23 ± 12	51 ± 39*	30 ± 11	0.013
Total cholesterol (mmol/L)	5.3 ± 0.7	5.1 ± 1.2	4.7 ± 1.4	0.342
Triglycerides (mmol/L)	1.7 ± 0.9	1.5 ± 0.8	1.3 ± 1.1	0.328
Medications				-
Statins	0	4	10	
Blood pressure	0	1	8	
Metformin	0	0	12	

Data are means ± SD.

*Significant difference disease vs. control (p < 0.05).

^†^Significant difference Type 2 diabetes vs. NAFLD (p < 0.05).

VO_2peak_, peak oxygen consumption; ALT, alanine aminotransferase.

### Experimental protocol

All participants were screened with resting blood pressure measurements (Suntech Tango+, Suntech Medical Ltd, Oxford), maximal cardiopulmonary stress testing with a 12-lead ECG (Custo med GmbH, Ottobrunn, Germany) and height and weight measurements using a stadiometer and electronic scale respectively (SECA, Birmingham, UK). Following screening, participants underwent fasting blood measurements. All study participants then underwent a magnetic resonance protocol including cine imaging, cardiac tagging, and phosphorus cardiac spectroscopy, hepatic spectroscopy and visceral fat analysis performed during a single session. Written informed consent was obtained from each participant. The study protocol was approved by Newcastle and North Tyneside 1 Research Ethics Committee.

### Fasting blood measures

Fasting blood samples were analyzed for whole blood glucose (YSI 2300 Stat Plus-D, Yellow Springs Instruments, Yellow Springs, OH). ALT, total cholesterol, triacylglycerols and HbA_1c_ were analysed in a clinical pathology accredited laboratory (Newcastle Upon Tyne Hospital NHS Foundation Trust, Department of Clinical Biochemistry) in both patient groups.

### Cardiac cine imaging

A 3 T Philips Intera Achieva scanner (Best, NL) was used for cardiac examinations.

**Figure 1:**
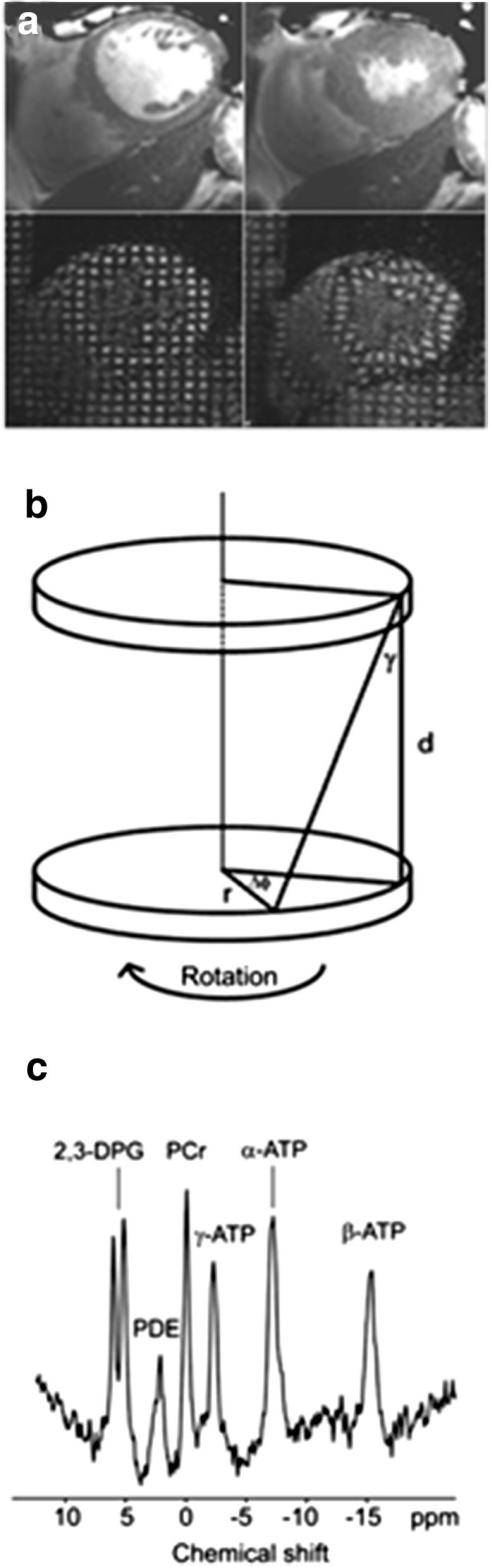
**Cardiac MRI techniques.** These include **(a)** Cardiac cine imaging (top) and cardiac tagging (bottom) at diastole (left) and systole (right), showing how a rectangular grid of nulled signal applied at diastole remains with the tissue through the cardiac cycle, allowing calculation of strain and torsion. **(b)** Tagging in two parallel sections allows the calculation of the torsion (the longitudinal-circumferential shear angle ϒ) between two short-axis planes a distance *d* apart with radius *r* where one short-axis plane rotates through ΔΦ relative to the other. ϒ = tan^−1^[(2*r* sin(ΔΦ/2))/*d*]. **(c)** Phosphorus spectroscopy from a control subject (PCr/ATP = 1.95). Spectrum presented before correction for saturation due to blood content, flip angle at the cardiac tissue and heart rate.

A dedicated six-channel cardiac coil (Philips) was used with the participants in a supine position and ECG gating. Short-axis balanced steady-state free precession images were acquired covering the left ventricle (FOV = 350 mm, TR/TE = 3.7/1.9 ms, acceleration factor 17, flip angle 40°, slice thickness 8 mm, 0 mm gap, 14 slices, 25 phases, resolution 1.37 mm). Analysis was performed using view forum workstation (Philips) on short axis slices at end-diastole and end-systole (Figure 1a). Details of our algorithms for calculating left ventricular mass, wall thicknesses, blood pool volumes and measures of systolic and diastolic function have been previously published [24]. The eccentricity ratio, a measure of concentric remodelling, was calculated by dividing left ventricular mass by end-diastolic blood volume.

Preload, afterload, contractility, and ventricular-arterial coupling were calculated from this data in combination with blood pressure measurements. Preload was determined by the end-diastolic volume, afterload by arterial elastance [(E_a_) = end-systolic pressure (systolic blood pressure x 0.9)/stroke volume (normalized to body surface area)], contractility by end-systolic elastance [(E_es_) = end-systolic pressure/end-systolic volume (normalized to body surface area)], and ventricular-arterial coupling by the ratio of E_es_/E_a_.

### Cardiac tagging

Cardiac tagging works by applying radiofrequency pulses to cancel magnetic resonance signal from the myocardium in diastole in a rectangular grid pattern and tracking the deformation of these tags through the rest of the cardiac cycle (Figure 1a + b). A turbo field echo sequence with acceleration factor 9 was used to collect short axis slices (TR/TE/FA/NEX =4.9/3.1/10^0^/1, SENSE factor 2, FOV 350x350mm, voxel size 1.37 mm, tag spacing 7 mm). The detailed protocol and analysis procedure to derive cardiac circumferential strain, torsion and the torsion to shortening ratio have been previously published [22].

### Cardiac spectroscopy

Cardiac high-energy phosphate metabolism was assessed using ^31^P-MRS (Figure 1c). With participants lying in a prone position with their heart at magnet isocentre, data was collected using a 10 cm diameter ^31^P surface coil (Pulseteq, UK) for transmission/reception of signal. A slice selective, cardiac-gated cardiac gated 1-dimensional chemical shift imaging (1D-CSI) sequence was used to eliminate contamination from the liver, with spatial pre-saturation of lateral skeletal muscle to avoid spectral contamination. Sixteen coronal phase-encoding steps were used, yielding spectra from 10-mm slices (TR = heart rate, 192 average, approx. 20-min acquisition time). Spectral locations were overlaid onto an anatomical image and the first spectrum arising entirely beyond the chest wall was selected. Details of our processing and correction of the spectra for blood contamination, saturation and excitation flip angle have been published previously [22].

### Liver and visceral fat measurement

Intrahepatic lipid was measured by localized T2-corrected ^1^H-magnetic resonance spectroscopy at multiple echo times using the point-resolved spectroscopy (PRESS) sequence: TR/TEs = 3000 ms/36,50,75,100,125,150 ms, voxel size 3x3x3cm, placed in the posterior right lobe to avoid major vessels. Visceral fat content was performed by acquiring images at the L4/L5 junction using a three-point Dixon sequence (TR/TE/number of averages/flip angle 50 ms/3.45, 4.60, 5.75 ms/1/5°, matrix 160×109, median field of view (FOV) 440 mm, range 400–480 mm to suit subject size with 70% phase FOV). Details of liver and visceral fat analysis have been previously published [25].

### Statistical analysis

Data are presented as means ± SD unless otherwise stated. All statistical tests were two-sided and performed using SPSS version 19 (IBM, NY, US). Continuous data were tested for normality using the Sharipo-Wilk test. Between group differences were evaluated using a one-way ANOVA with Bonferroni correction methods for multiple comparisons and a non-parametric alternative (Kruskal Wallis) for non-normally distributed data. Spearmans rank correlation was used to observe any relationship between metabolic parameters and cardiac parameters. Any significant relationships were then entered into a multiple linear regression model, adjusting for age, systolic blood pressure and anthropometry (body mass index (BMI), body surface area, weight, systolic blood pressure). The goal of these analyses was to determine which factors were responsible for the differences in cardiac structure and function between groups. P values <0.05 were considered statistically significant.

## Results

Table 1 summarises the demographic data of the three groups. Body weight, BMI and systolic blood pressure were significantly higher in Type 2 diabetes and NAFLD compared with controls (p < 0.05). Both NAFLD and Type 2 diabetes demonstrated increased liver fat (9.4 ± 4.3 *vs.* 7.9 ± 6.7 *vs.* 2.5 ± 0.9%; p < 0.05) while fasting glucose was higher in Type 2 diabetes only (7.2 ± 1.4 mmol/L; p < 0.05). Peak oxygen consumption (VO_2peak_) was significantly lower in the Type 2 diabetes group compared to NAFLD (p < 0.01). There was no significant difference in visceral adipose tissue (p = 0.120), blood cholesterol (p = 0.342) or triglycerides between patients and controls (p = 0.328, Table 1), however it should be noted that both Type 2 diabetes and NAFLD patients were taking lipid lowering medication.

### Cardiac structure and systolic function

**Figure 2:**
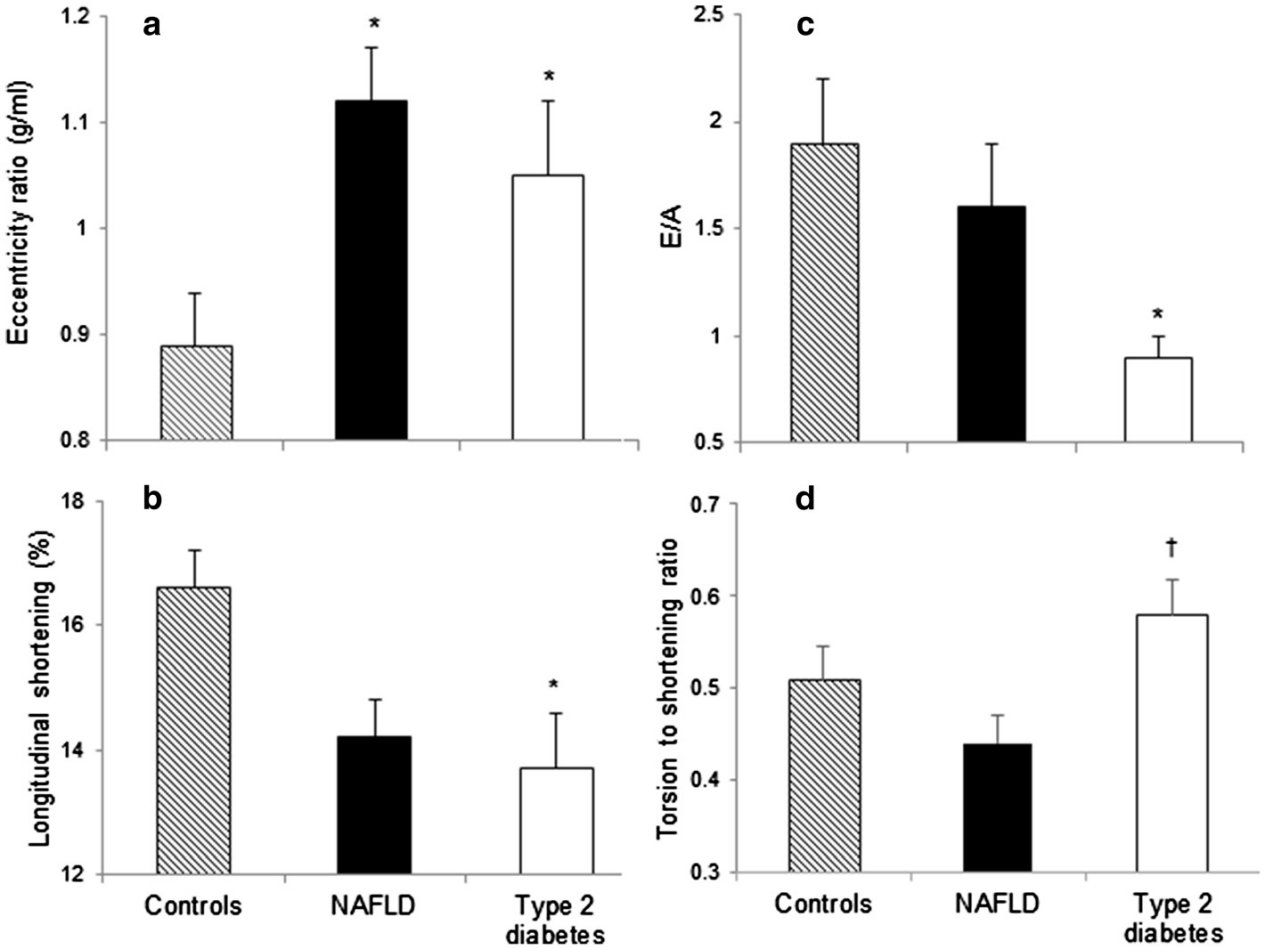
**Measures of cardiac structure and function. (a)** eccentricity ratio, **(b)** longitudinal shortening **(c)** E/A and **(d)** torsion to shortening ratio, in control, NAFLD and Type 2 diabetes adults. Data are means ± SE.*Significant difference disease vs. control (p < 0.05). †Significant difference Type 2 diabetes vs. NAFLD (p < 0.05).

Left ventricular mass was similar in all groups (p = 0.581). The NAFLD group demonstrated thicker walls at end-systole and end-diastole (p < 0.05). Cardiac structural concentric remodelling was observed in both NAFLD and Type 2 diabetes, as shown with an increased eccentricity ratio (1.12 ± 0.2 *vs*. 1.05 ± 0.3 *vs.* 0.89 ± 0.2 g/ml; p < 0.05) and reduced end-diastolic volume indexed when compared with healthy controls (p < 0.05) (Table 2, Figure 2a). An increased eccentricity ratio was associated with diastolic dysfunction in NAFLD (E/A: r = −0.4, p = 0.05) and Type 2 diabetes (E/A: r = −0.56, p = 0.012; Early filling rate: r = −0.59, p = 0.009; Early filling %; r = −0.64, p = 0.01) but not in the control group.

**Table 2:** **Magnetic resonance imaging measurements of cardiac structure, function and metabolism**

	**Controls**	**NAFLD**	**Type 2 diabetes**	**P value**
	**(n = 19)**	**(n = 19)**	**(n = 19)**	
**Cardiac structure**				
Left ventricular mass (g)	102 ± 26	114 ± 31	108 ± 28	0.581
Left ventricular mass indexed (g/m^2^)	55 ± 12	59 ± 11	53 ± 12	0.273
Wall thickness diastole (mm)	7 ± 1	8 ± 1*	6 ± 2*^†^	0.000
Wall thickness systole (mm)	12 ± 2	14 ± 3*	12 ± 3	0.016
Eccentricity ratio (g/ml)	0.89 ± 0.2	1.12 ± 0.2*	1.05 ± 0.3*	0.004
End-diastolic volume indexed (ml/m^2^)	64 ± 18	54 ± 14	52 ± 14	0.039
End-systolic volume indexed (ml/m^2^)	27 ± 9	21 ± 9	21 ± 10	0.63
**Systolic function**				
Heart rate (bpm)	59 ± 9	61 ± 9	65 ± 9	0.178
Stroke volume (ml)	70 ± 19	64 ± 12	64 ± 17	0.437
Stroke index (ml/m^2^)	38 ± 10	33 ± 5	31 ± 7*	0.034
Cardiac output (L/min)	4.0 ± 0.8	3.8 ± 0.6	4.0 ± 0.9	0.754
Ejection fraction (%)	59 ± 5	63 ± 8	61 ± 10	0.332
Longitudinal shortening (%)	16.6 ± 2.8	14.2 ± 2.7	13.7 ± 4*	0.017
Arterial elastance	3.32 ± 0.85	4.07 ± 0.78*	4.38 ± 1.05*	0.004
Ventricular elastance	5.04 ± 2.05	7.62 ± 3.22*	7.72 ± 4.08*	0.011
Ventricular-arterial coupling	1.50 ± 0.35	1.82 ± 0.60	1.75 ± 0.72	0.263
**Diastolic function**				
Early filling percentage (%)	69 ± 11	65 ± 11	57 ± 9*	0.003
E/A	1.9 ± 1.4	1.6 ± 1.3	0.9 ± 0.4*	0.015
Early diastolic filling rate (ml/s)	312 ± 121	265 ± 95	244 ± 76	0.105
Late diastolic filling rate (ml/s)	203 ± 73	212 ± 70*	288 ± 99*	0.009
**Strain and torsion**				
Peak endocardial circumferential strain (%)	22 ± 5	28 ± 4*	24 ± 5^†^	0.001
Peak whole wall circumferential strain (%)	17 ± 3	19 ± 2	16 ± 4^†^	0.012
Peak torsion (°)	6.6 ± 1.8	6.9 ± 2.2	8.0 ± 2.5	0.127
Torsion recoil rate (%/ms)	0.25 ± 0.12	0.17 ± 0.12	0.27 ± 0.1^†^	0.017
Torsion to shortening ratio	0.51 ± 0.15	0.44 ± 0.13	0.58 ± 0.16^†^	0.019
**Metabolism**				
PCr/ATP ratio	1.9 ± 0.3	1.8 ± 0.3	1.8 ± 0.3	0.543

Data are means ± SD.

*Significant difference disease vs. control (p < 0.05).

^†^Significant difference Type 2 diabetes vs. NAFLD (p < 0.05).

PCr/ATP, phosphocreatine/Adenosine triphosphate.

Systolic function was impaired in the Type 2 diabetes group, evidenced by a lower stroke index (31 ± 7 *vs.* 38 ± 10 ml/m^2^; p < 0.05) and reduced longitudinal shortening (13.7 ± 4 *vs.* 16.6 ± 2.8%; p < 0.05) when compared to controls (Figure 2b). There were no differences in heart rate, stroke volume, cardiac output and ejection fraction between groups and no significant correlations between measures of structural parameters and systolic function with fasting glucose or HbA_1c_. Arterial elastance (afterload) and ventricular elastance (ventricular stiffness) were both increased in NAFLD and Type 2 diabetes compared to controls (p < 0.05) but the ratio between the two (ventricular-arterial coupling) was not different between groups (Table 2).

### Diastolic function

**Figure 3:**
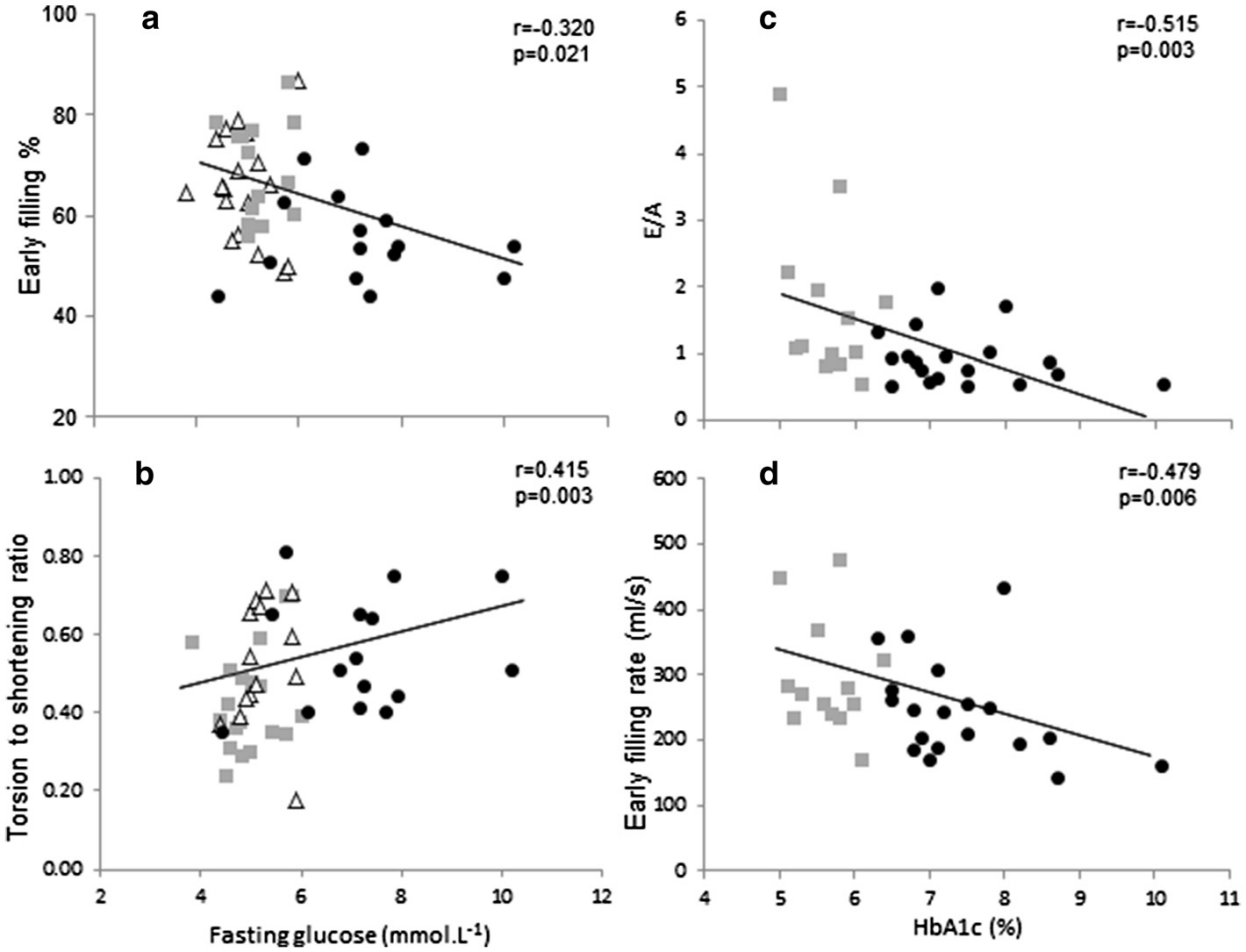
**Associations between glycemic control and measures of cardiac function.** *Triangle = Control, Square = NAFLD, Circle = Type 2 diabetes.* Relationships between **(a)** fasting glucose and early filling percentage, **(b)** fasting glucose and torsion to shortening ratio, **(c)** HbA_1c_ and E/A and **(d)** HbA_1c_ and early filling rate, are presented in the figure. HbA_1c,_ haemoglobin A_1c_.

The early to late filling ratio (E/A) was significantly lower in Type 2 diabetes compared to controls (0.9 ± 0.4 *vs* 1.9 ± 1.4; p < 0.05) (Figure 2c) along with a decrease in early filling percentage (p < 0.05).The NAFLD group showed no significant change in these parameters compared to controls. Both the NAFLD and Type 2 diabetes groups displayed significant increases in late diastolic filling rate compared to controls (212 ± 70 *vs.* 288 ± 99 *vs.* 203 ± 73 ml/s; p < 0.05). Across the three groups, there was a moderate negative correlation between fasting glucose and early filling percentage (r = −0.32, p = 0.021) (Figure 3a). In addition, increased HbA_1c_ was associated with impaired E/A (r = −0.52, p = 0.003) and reduced early filling rate (r = −.48, p = 0.006) (Figure 3c + d). When controlling for the baseline differences in age, systolic blood pressure and anthropometry across groups, glucose (β = −0.26, p < 0.05) remained a significant predictor of early filling percentage. Age was a significant predictor of early filling percentage (β = −0.5, p < 0.01), E/A (β = −0.66, p < 0.01) and early filling rate (β = −0.48, p < 0.01).

### Cardiac torsion and strain

Cardiac torsion and strain differed across groups, with higher endocardial circumferential strain in the NAFLD group and elevated torsion in the Type 2 diabetes group, as shown by the significantly higher torsion to shortening ratio in Type 2 diabetes compared to NAFLD (0.58 ± 0.16 *vs.* 0.44 ± 0.13, p < 0.05, Figure 2d). Peak torsion was 24% greater in the Type 2 diabetes group compared to controls. Fasting glucose was moderately correlated with the torsion to shortening ratio (r = .42, p = 0.003) (Figure 3b). When controlling for baseline differences in age, systolic blood pressure and anthropometry, the association between fasting glucose and the torsion to shortening ratio was approaching significance (β = 0.25, p = 0.072).Similarly, age was approaching significance in predicting the torsion to shortening ratio (β = 0.29, p = 0.057).

### Cardiac metabolism

There was no difference in phosphocreatine/adenosine triphosphate (PCr/ATP) amongst NAFLD and Type 2 diabetes adults compared to controls (1.8 ± 0.3 *vs.* 1.8 ± 0.3 *vs.* 1.9 ± 0.3; p = 0.543) and PCr/ATP correlated with no measures of cardiac structure or function.

## Discussion

This is the first study to compare the effect of Type 2 diabetes and NAFLD on cardiac structure, function and metabolism using the most sensitive cardiac magnetic resonance techniques. The major findings suggest that despite similar levels of concentric remodelling, individuals with Type 2 diabetes demonstrate significantly greater diastolic and subendocardial dysfunction in comparison with NAFLD and healthy adults. There were significant relationships between glycaemic control and measures of cardiac function, suggesting that hyperglycemia itself is an important factor contributing to these sub-clinical cardiac changes.

The results indicate concentric remodelling in both NAFLD and Type 2 diabetes independent of changes in left ventricular wall mass. Using similar magnetic resonance techniques, Diamant et al. [12] also failed to demonstrate an increase in left ventricular wall mass in adults with Type 2 diabetes which is in contrast to studies using echocardiography [26]. Echocardiography has however been found to overestimate cardiac size [27]. In both NAFLD and Type 2 diabetes, an increased eccentricity ratio was associated with diastolic dysfunction. We speculate that the reduced left ventricular cavity may impair diastolic filling. The NAFLD group display more prominent structural changes than the Type 2 diabetes group, with thicker walls at diastole and systole. This enables the left ventricle to generate increased force and pressure thereby preserving systolic function [28].

The data show left ventricular diastolic and systolic dysfunction in Type 2 diabetes but not NAFLD, despite equivalent degrees of blood pressure and concentric remodelling. Early diastolic filling due to ventricular relaxation accounts for roughly 80% of ventricular end-diastolic volume in a young healthy heart and declines with age [29]. Impaired early filling observed in the present study indicates greater myocardial stiffness. Diastolic dysfunction is an independent predictor of mortality [30] which warrants the need for therapies to target these preclinical cardiac changes. A recent longitudinal study demonstrated no decline in diastolic dysfunction after 6 years of follow up in adults with Type 2 diabetes, when cardiovascular risk factors (such as blood pressure, BMI and blood glucose) were managed [13]. Interventions targeting these risk factors therefore need to be a priority in the treatment of diastolic dysfunction. The present study uses MRI which is in contrast to the aforementioned study and many other reports in the literature. MRI provides a robust non-invasive assessment of cardiac function and is considered the gold standard for measures of cardiac structure, enabling the measurement of cardiac changes in a unique non-invasive way.

Analysis of cardiac deformation by 3D magnetic resonance tagging demonstrated a raised torsion to shortening ratio in Type 2 diabetes with peak torsion 24% greater in this group. A decrease in circumferential and longitudinal strain accompanied with a rise in torsion has been previously reported in Type 2 diabetes [15]. The torsion to shortening ratio is a marker of the dominance of subepicardial fibres exerting their effects over the subendocardium [31]. Torsion is a normal feature of cardiac contraction and results in a counter clockwise twisting motion when viewed from the apex to base. Subepicardial fibres act over a larger radius, therefore, during contraction they are dominant over subendocardial fibres which partially counteract this twisting motion [31]. The relative dysfunction of the subendocardium in Type 2 diabetes, manifested as an increase in torsion could be attributed to subendocardial fibrosis [31]. Subendocardial dysfunction reduces longitudinal shortening as demonstrated in this study. In contrast to Type 2 diabetes, NAFLD participants demonstrate increased strain and maintained torsion when compared to controls. The large difference in radii between epicardial and endocaridal fibres (due to both increased wall thickness and reduced end-diastolic blood volume indexed) in NAFLD, means endocardial strain has to increase for torsion to be maintained.

**Figure 4:**
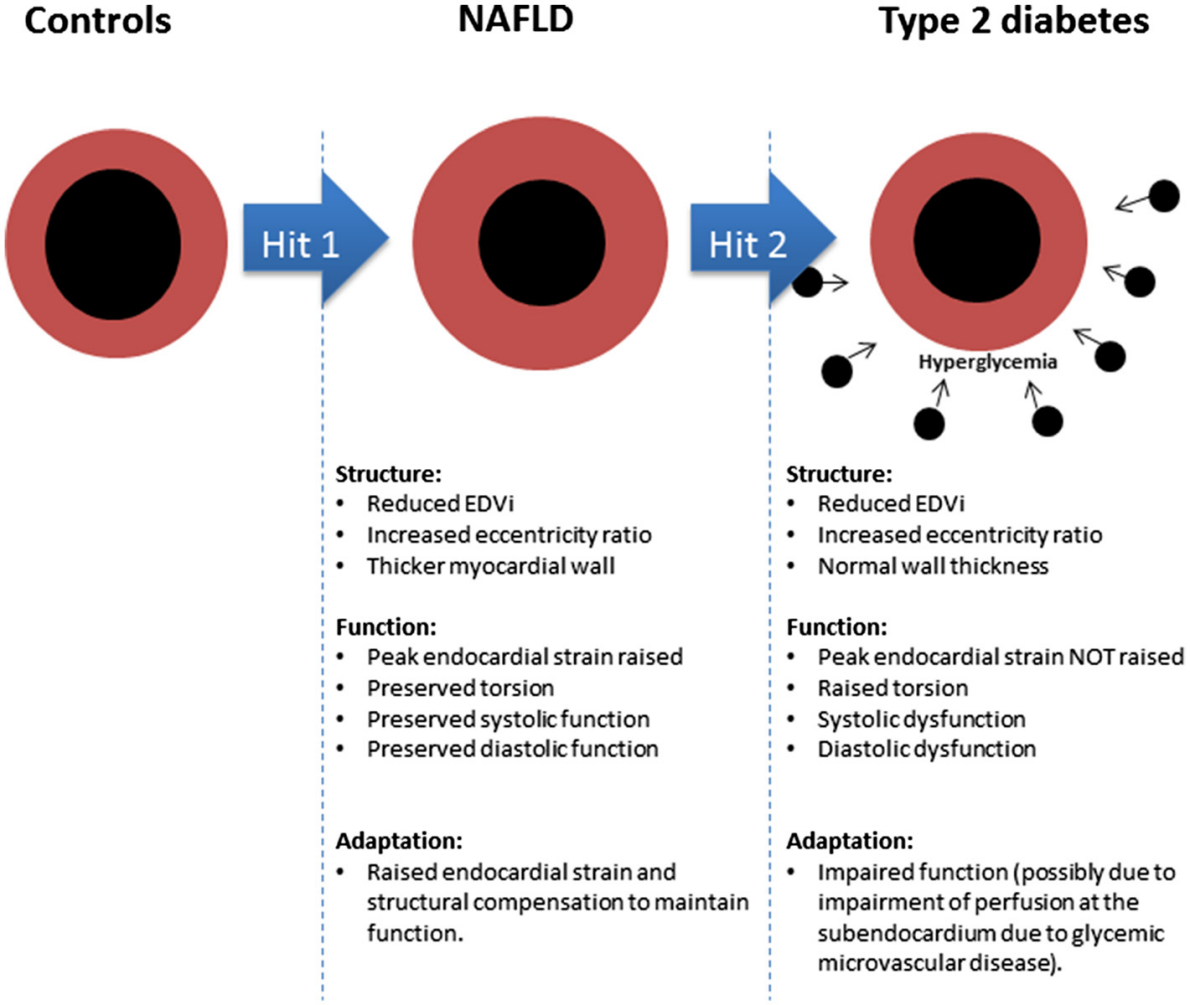
**Postulated interactions between cardiac parameters across controls, NAFLD and Type 2 diabetes.**

Across the three groups, age and fasting blood glucose were predictors of the changes in cardiac function. It has been previously demonstrated that age is associated with diastolic dysfunction and an increase in the torsion to shortening ratio [29,31,32], and our results confirm these findings. Despite this, fasting blood glucose independent of age, influenced cardiac function, suggesting that metabolic disease exaggerates the ageing phenotype. The relative contribution of liver fat and other metabolic parameters (BMI, Systolic Blood Pressure, Total Cholesterol, Triglyceride) with cardiac complications is an important question. In the linear regression model, there were no consistent predictors of cardiac dysfunction other than blood glucose. However, to fully explore this question larger studies are required. The relationship with blood glucose is of interest. We speculate that NAFLD participants who have high liver fat with stable blood glucose demonstrate higher endocardial strain and structural compensation to maintain cardiac function. The progression to Type 2 diabetes which is characterised by high blood glucose may lead to endocardial damage, resulting in impaired function. This is reflected in the significant relationship between blood glucose and measures of cardiac function across the three groups. Preventing a rise in blood glucose should therefore be a priority in the clinical management of NAFLD. The postulated interactions between cardiac parameters in NAFLD and Type 2 diabetes are summarised in Figure 4.

It has been previously demonstrated that poor glycemic control is associated with an increased risk of heart failure in adults with Type 2 diabetes [33]. The present study builds on this by demonstrating an association between glycemic control and changes in myocardial function. The relationship between cardiac torsion and glycemic control has not been previously shown and only a few studies have demonstrated a relationship between glucose control and diastolic function [11,16]. Direct primary effects on the myocardium or secondary effects on peripheral resistance (afterload) are two pathways in which blood glucose could interfere with diastolic function. This is because diastolic distensibility (raised diastolic pressure at any level of diastolic volume) can arise from altered myocardial elastic properties (fibrosis) or prolongation of ventricular relaxation [34]. Raised afterload slows ventricular relaxation which can alter the pressure gradient required during early diastolic filling and elevations in afterload have been shown to induce left ventricular diastolic dysfunction [35]. In this study, arterial elastance (afterload) was increased in Type 2 diabetes and NAFLD but was not associated with glucose control. This is suggestive of a direct impact of glucose on the myocardium rather than peripheral resistance.

Despite changes in structure and function, cardiac high energy phosphate metabolism was similar between the three groups. A PCr/ATP ratio reduction of 35% has previously been demonstrated in participants with Type 2 diabetes [36] and a 13% reduction in NAFLD, compared to healthy controls [37]. The reduction in PCr/ATP ratio has also been correlated with diastolic dysfunction in people with Type 2 diabetes leading the authors to postulate increased concentration of NEFA in metabolic disease causes a switch from glucose to lipid cardiac metabolism, reducing efficiency of ATP production and causing cardiac functional changes [12]. However, the characteristics of the Type 2 diabetes participants in the aforementioned study were different from those in the present study. Specifically, they were not taking lipid-lowering medication and therefore had higher levels of triglyceride and total cholesterol values. Indeed, when participants were following current NICE guidelines [38] and using lipid-lowering medications, the PCr/ATP ratio between Type 2 diabetes and controls were comparable [11]. In the present study, triglyceride and cholesterol levels were similar in Type 2 diabetes, NAFLD and controls which could explain the lack of secondary difference in cardiac metabolism. These data also suggest that changes in high energy phosphate metabolism may reflect differences in substrate oxidation/supply rather than an underlying metabolic defect in the myocardium.

Limitations of the study should be considered. The cross sectional nature does not allow insight into causality of the abnormalities identified. Hypertension is a common comorbidity in metabolic disease which complicates the distinction of the separate impact of glucose control and high blood pressure on cardiac function. However, blood glucose had a significant relationship with measures of cardiac function independent of blood pressure. The present manuscript focused on functional and metabolic changes but does not measure perfusion or steatosis, two mediators of metabolism and function, due to the duration of MRI scans and tolerability by patients. Myocardial triglyceride accumulation is an early sign of heart disease in Type 2 diabetes and is associated with changes in cardiac function, in particular diastolic dysfunction [16,39,40]. The present data reinforces the need for further exploration of the interrelationship between, glycemic control, cardiac function/metabolism, perfusion and steatosis. In addition, we were unable to assess stress MRI meaning cardiac abnormalities have only been identified at rest.

## Conclusions

In summary, changes in cardiac structure are evident in adults with Type 2 diabetes and NAFLD without overt cardiac disease and without changes in cardiac energy metabolism. The growing prevalence of metabolic disorders puts large numbers at risk of these underlying cardiac changes. Only the Type 2 diabetes group display diastolic and subendocardial dysfunction and glycemic control may be a key mediator of these cardiac changes. Managing blood glucose should therefore be a priority for clinical care teams to prevent cardiac complications in adults with Type 2 diabetes and NAFLD.

## Abbreviations

NAFLD: Non-alcoholic fatty liver disease
GGT: Serum γ-glutamyltransferase
ALT: Alanine aminotransferase
AST: Aspartate aminotransferase
HbA_1c_: Glycated hemoglobin
VO_2peak_: Peak oxygen consumption
E/A: Early to late filling ratio
PCr/ATP: Phosphocreatine/adenosine triphosphate
BMI: Body Mass Index
MRI: Magnetic resonance imaging
